# Sulphate of Cinchonidia

**Published:** 1877-10-20

**Authors:** C. G. Polk


### SULPHATE OF CINCHONIDIA.

By C. G. Polk, M. D.

The increase in the price of the sulphate of quinia has almost imposed a prohibition upon its employment, and naturally turns the attention of the physician to a substitute less costly. Of the various articles which have been tested, none except the sulphate of Cinchonidia have, so far, given general satisfaction. The price, not exceeding half the cost of quinia, and the beneficial results following its administration approaching so nearly the more costly alkaloid, that physicians have learned to employ it in all cases in which the pecuniary consideration is important,

As an anti-periodic I have employed it with a very high degree of satisfaction in the following formula:

R. Sulph. cinchonidia........grs.xv.
Fluid ext. gelseminum.... gtt.xx.
Fluid ext. glycyrrh........dr.iii.
Syrup aurant cort..........driv.
Mucil acaciae qs. ad.... ...oz.ii. M.
S. Desertspoonful every hour until taken.

During the past summer, I have used it in a dozen cases of intermittent fever, without a single failure. In only one case was there a paroxysm after taking the above quantity, and none in which a second paroxysm occurred. The taste is not at all disagreeable, the cinchonidia scarcely imparting a trace of its bitter. Children take it without inconvenience, and, it seems to me, it is less liable than the quinia salt to derange the brain or stomach.

In remittent fever I have employed the same formula, breaking up the disease in three or four days.

I have also employed it in five cases of enteric fever, with large doses of sulphuric acid, and they were all convalescing by the twelfth day. In fact, I have learned to regard it every way equal to the more costly quinia, and as fast as I can control my long acquired fondness for the latter, am I evincing my firm conviction of its value, and employing it in my prescriptions.

I throw out these suggestions, hoping that the additional grain of evidence may aid in the general use of this valuable medicine.

The only real rival it has is the cincho-quinia, and I think there is a large balance of advantage in favor of the cinchonidia. I think consciencious analyses of cincho-quinia have determined different results, and I think variation of constituents inseparable from a preparation embodying all the constituents of cinchona bark.

The variation in strength is an objection against the cincho-quinine, its comparative freedom from bitterness is an item in its favor. I have heard physicians object to it on ground of its proprietary character.

But the relative merits of the two can not be determined elsewhere than at the bedside of the sick. One clinical fact is worth ten thousand speculations.

The great question of the physician is, what is best to heal the sick? This determined, he should use the best, regardless of every other consideration. Of course, officinal preparations should, other things being equal, be preferred. Patented preparations should be rejected, unless the exact composition be known, and then it is better to designate the articles and quantity rather than favor a nostrum.

I think, then, surveying the subject in both its ethical and therapeutical light, there is every reason to give preference to sulphate of cinchonidia. The statement recently made by Dr. Ferdinand King, in THE SOUTHERN MEDICAL RECORD, that it requires three times as much cinchonidia as quinia, is very contrary to my experience. I have obtained as good results from the cheaper alkaloid by increasing the amount one-fourth, as I have from quinia sulphate.

I do not deny that cincho-quinine is a good preparation, I believe it is; but I am very positive that sulphate of cinchonidia is a perfect substitute for sulphate of quinia.

It is believed that both cincho-quinine and Stearns' sweet quinia consists largely of pure cinchonia. The latter pure cinchonia (we do not now speak of the sulphate of cinchonia, but of the alkaloid itself,) has not, perhaps, received the attention that it deserves. By mixing twelve (12) parts of this with sixty (60) of sugar of milk, and adding one (1) part only of bi-carbonate of soda, a powder can be prepared which will readily mix with a small quantity of milk or cream, and forms a tasteless compound, and thus desirable to use in many cases. The administration of it should then be followed by a glass of water, and after an interval of half hour by acidulous drinks, when all the effects of quinia will be produced.

